# How to Support Participants in Urogenital and Prostate Clinical Trials: A Qualitative Perspective

**DOI:** 10.1002/pon.70214

**Published:** 2025-07-14

**Authors:** Natasha A. Roberts, Ian D. Davis, David Wyld, Jeffrey Goh, Maxwell Thompson, Craig Gedye, Haryana M. Dhillon

**Affiliations:** ^1^ Metro North Health Service Brisbane Australia; ^2^ The University of Queensland Brisbane Australia; ^3^ Queensland University of Technology Kelvin Grove Australia; ^4^ ANZUP Cancer Trials Group Sydney Australia; ^5^ Monash University Melbourne Australia; ^6^ Eastern Health Melbourne Australia; ^7^ ICON Cancer Care Adelaide Australia; ^8^ The University of Sydney Sydney Australia

**Keywords:** cancer, clinical trials, health services, oncology, patient experience, qualitative research, supportive care

## Abstract

**Background:**

Clinical trials are fundamental to improving cancer treatment and clinical outcomes. Whilst clinical trials offer treatment options to affected individuals, less is known about how to best support those who participate.

**Aim:**

To explore the experiences of participants in urogenital and prostate cancer clinical trials to identify opportunities to improve care for this cohort of patients.

**Methods:**

This qualitative study was conducted in Australian hospitals. Participants were included if they had consented to participate in an Australian collaborative investigator led clinical trial for prostate or other urogenital cancer. Semi‐structured interviews with participants were completed by telephone. Using verbatim transcriptions, their experiences relative to key stages of a clinical trial were analysed using a Framework Method. Findings were interpreted to develop key recommendations for use by investigators developing a clinical trial.

**Results:**

22 people consented and participated in interviews. They came from rural, regional, and metropolitan areas across Australia. Participants were being treated for advanced uro‐genital and prostate cancers. Across interviews, participants spoke about their supportive care needs and how they valued their relationships with the multi‐disciplinary research team and the clinical oncology and urology teams. There was variation in participant needs according to the stage of the clinical trial. Despite times of uncertainty, it was reported clinical trial participation was meaningful. Receiving information about overall findings of the clinical trial was important to participants allowing them to understand the impact of their contribution.

**Conclusion:**

Understanding the experiences of clinical trial participants can improve the design of trial protocol processes and procedures. Participants want to stay connected with their clinical trial.

## 1 Background

Clinical trials are fundamental to improving cancer treatment and outcomes, forming an essential part of cancer research [1]. Research focussing on urogenital and prostate cancers in particular, has increased in the last 10 years, with significant improvements in treatment options and health outcomes for these populations, including survival and/or quality of life [2, 3].

Patient experience is a recognised dimension in the assessment of health care quality assessment [4], participation in clinical trials is part of healthcare, rendering it important to understand and respond to patient experience. Investigating patients' experiences provides informative context to what is known about illnesses, treatment, and health care systems. A comprehensive evidence base of patient/participant experience within urogenital and prostate cancer clinical trials is needed to ensure the delivery of quality care [5, 6, 7].

Whilst there is an evidence base about the experiences of patients and families in clinical trials [6, 7], and there has been investment by the clinical trials industry to engage with patients in clinical trial design [8], optimisation of patient experience is still a pressing need [9, 10]. Requirements from patients vary across the stages of a clinical trial, from screening to survival follow up [11]. In Australia, there has been an increased interest in patient experience of clinical trials as hospitals now have mandatory accreditation requirements for clinical trials.

The Australian and New Zealand Urogenital and Prostate (ANZUP) Cancer Trials Group is an internationally recognised collaborative cancer trials group with strong engagement with academic, industry, not‐for‐profit, and health sectors. Its ethos is to bring together all clinical disciplines, researchers, and community advocates involved in urogenital and prostate cancer research and treatment to conduct investigator‐initiated clinical trials addressing questions of importance to patients and clinicians. The group has a portfolio of investigator‐led clinical trials across bladder, kidney, urothelial, penile, testicular, and prostate cancers [3]. A consumer advisory panel (CAP) is integral to the core business of ANZUP, providing input from the early stages of clinical trials concept development, through to dissemination of findings and translation into clinical practice.

Participation in an ANZUP clinical trial is unique as it is investigator‐led and may offer an additional treatment pathway for patients in the context of a traditional cancer treatment centre [12, 13]. These trials designs will often align closely with standard of care procedures. There is little understanding of the experiences of investigator led urogenital prostate cancer trials across the stages of a clinical trial, from screening to survival follow‐up. Motivated to take an opportunity to better improve the health experience of patients and families, we aimed to answer the following research question: *What is the experience of participating in an ANZUP clinical trial, taking into consideration the stage of the trial including screening, consent, treatment and post‐treatment follow‐up?*

## 2 Methods

This was a qualitative study. Three hospitals across Australian state health services participated over 12 months in 2019 and 2020. Ethical approval was granted by the Royal Brisbane and Women's Hospital Ethics Committee (HREC/18/QRBW/251) and all participants provided written informed consent. Reporting has followed Consolidated Criteria for Reporting Qualitative Research (See Supporting Information S1).

A semi‐structured interview guide was developed using the work of Manson et al. [11] to explore questions specific to stages of a clinical trial, including: screening; consent; study commencement; during intervention/treatment; end of treatment; and ongoing study follow up (See Supporting Information S2). The sole inclusion criterion was prior consent for an ANZUP clinical trial. At each site, when a study opened, local research teams identified all ANZUP study participants attending appointments and flagged them to clinical teams to discuss this study, providing them with the participant information statement. This process continued until study recruitment was closed. If participants consented to be contacted, a member of the study team telephoned them to discuss participation. The signed consent form was returned to the research team via electronic or postal mail, who then contacted potential participants to schedule an interview.

Semi‐structured interviews were completed by NR by telephone and audio recorded, with a duration of 45–75 min. Interviews were transcribed and de‐identified by a professional transcriber independent of the study. Initial coding commenced after two participants completed interviews, with further coding occurring iteratively after every five participants until no new themes were identified.

Memos were made after interviews and regularly during analyses. All participants were offered the opportunity to review their transcripts, and eight provided further comments. Data were analysed using the Framework Method, which has its foundations in Grounded Theory [14]. This approach was chosen to inductively identify participant experiences. Data analysis was performed by four member of the research team who were gender, age, and professional group diverse (NR, HB, MT, HD). The research team consisted of two experienced, post‐graduate trained qualitative researchers (NR and HD), and novice qualitative researchers with undergraduate training in research methods (HB and MT). During the analysis the researchers familiarised themselves with the transcripts independently reading their designated transcripts and making notes. Codes were inductively identified using statements in the transcript and colour coded into word versions of the transcripts. Team members met to discuss transcripts and codes, grouping similar concepts to form the analytical framework. Member checking of transcripts and cross coding were used to ensure rigour.

The final findings were then discussed by the broader research team, and an interpretive analysis [15] applied to develop recommendations applicable to future clinical trials research.

## 3 Results

Of the 22 people approached, all consented to be contacted and signed written consent. Of those, one did not proceed to an interview for health reasons. Participants were from a range of geographical locations, 11 regional, eight metropolitan, and three rural, across three Australian states: Victoria (*n* = 5), New South Wales (*n* = 2), and Queensland (*n* = 15). All participants reported having been diagnosed with an advanced cancer, including prostate (*n* = 13), renal (*n* = 8), and bladder cancer (*n* = 1). The age range was 52–71 years. There were 4 female and 18 male participants, five participants had been treated on a clinical trial for localised kidney cancer [16], four were seeking treatment for metastatic castration resistant prostate cancer [17, 18], six had received clinical trial treatment for metastatic prostate cancer [19, 20], and five participated in a clinical trial for metastatic or locally advanced kidney cancer [21, 22]. Of these, one participant was undergoing screening for trial participation, seven were on active treatment (the duration on the trial was not captured), and 13 were in follow‐up after treatment. None had previously participated in a clinical trial.

Findings were synthesised into key stages of a clinical trial including ‘Getting onto a Clinical Trial’, ‘Active Treatment’, ‘Life After Treatment’. Each is described below. The key concepts identified through data analysis are presented in Table 1.

**TABLE 1:** Key concepts identified as corresponding to stages of clinical trials.

Clinical trial stage	Key concepts	Definition	Quotations
Getting onto the trial *‘I'm feeling lucky’* *‘Trusting this is the right decision’*	Life and death	Perceptions on how a clinical trial can influence survival outcomes	*‘I'm hoping this study will give me a plan B’* PID22
	Screening	Experiences during investigations to assess trial eligibility	*‘Days of scans and tests and you don't even know what will happen, nothing is certain.’* PID8
	Trust	Trust in the clinicians who were involved with the referral to the clinical trial, or an investigator	*‘…so I got a call from my urologist…he said there's a trial’* PID7
	Informed consent	Reflections on the decision to participate	*‘I was very keen to get on…there was no decision to be made. It was a matter of get it. Try and get it at all costs’* PID16
	Understanding risks	Opinions about information relating to potential risks outlined in the consent process	*‘[he said]“no it's pretty routine. It's ok,” that sort of thing. So, he was pretty reassuring…he was pretty calm about it and he didn't seem [concerned], because the paperwork you get for the trial, the side‐effects are pretty comprehensive and makes it sound like they're likely to happen’* PID7
Active treatment *‘The comfort of structure’* *Relationships with the multi‐disciplinary team* *Developing measures of success*	Routine	The routines resulting from participation	*‘I have a blood test every Tuesday, treatment Thursday, scan every 8 weeks’* PID7
	Protocol	Perceptions protocolised treatment and assessments	*‘it was all pretty spelt out pretty quick clearly on what the treatment protocol was going to be’* PID20
	Structure	Experiences of the controlled clinical trial environment	*‘I think because of the trial I am on, those CT scans every 8 weeks, which is good to know that things are progressing in the right direction.’* PID7
	Timeliness	Timing of assessments, treatment, and appointments	*‘I think to some degree the waiting times are less…’* PID9
	Relationships	Relationships with the clinical trials team members	*‘It is the relationships that make you feel safe’* PID8
	Measuring success	Strategies taken to assess whether treatment was working	*‘I always go in with a lot of questions every time. I have an appointment with the oncologist now, every time. They go through my questions. So, I know what is going on’* PID9
	Toxicities	Experiences of treatment side effects	*‘You are doing well if you just suck it up….just focus on what you need’* PID8
Life after treatment *Finding ‘meaning’ from taking part in a clinical trial* *‘Staying close to the mother ship’*	Celebration and relief	Memories of finishing treatment	*‘it was nice to finish, not that I had too much trouble….I felt pretty good the whole way through. Was it a relief? It's a bit like finishing your exams…all think about when you're doing exams is the study and then doing the exams. All you think about is when you finish, when you finish, how good it will be’* PID6
	Making sense	How participants understand their experience	*‘It turns out I wasn't healthy, so its very good that I am on the trial. I was sceptical…at first’* PID7
	Giving back	Participant reflections on altruism and the contributions they make by taking part	*‘By giving my information, there may be someone just like me that I help’* PID12
	No regrets	Reflections on decisions to participate	*‘…why wouldn't you do a clinical trial? Absolutely if it's the best course of action..’* PID17
	Being an expert	Knowledge attained as a result of participation/increasing health literacy	*‘…you virtually know nothing to start with… you go along and you learn more as you go…’* PID14
	Monitoring	Experiences of ongoing study follow‐up	*‘but it's part of the process. Because if I don't have them, and the bastard inside me gets away from me, then we've got an issue. If I don't have blood tests, we don't know what's going on inside me. So, it's the necessary evil and you've just to take it in your stride…blood tests every 3 months’* PID12
	Trial results	Thoughts on wanting to know the outcomes of the trial	*‘I did catch up with my urologist who is at another hospital, he was saying the drug is now approved because of my trial’* PID17

*Note:* Results from matrix development during data analysis presenting the clinical trial stage, key concepts relating to that stage, a definition of this concept, and a exemplary quotation.

### 3.1 Stage 1: Getting on the Trial

#### 3.1.1 Theme 1.1: ‘I'm Feeling Lucky’

Participants described unique experiences of getting onto a clinical trial. Overwhelmingly, it was perceived as an opportunity not available to everyone. Being eligible was considered *‘lucky’* (PID22) with participation in a clinical trial described as an opportunity to disrupt an inevitable outcome.

I’d reached the point of running out of options to proceed…I was just very happy to proceed and see what chance I could get.

PID 9

But, screening assessments to determine eligibility made participants feel vulnerable. With uncertainty around whether individuals could *‘get on to the trial’* or whether they would be randomised to the experimental drug they were hoping for.

Massive relief. The most stressful part of my diagnosis, I reckon the whole thing, most stressful part was whilst I was waiting to find out if I got on. It was very exciting that I actually got on.

(PID16)

Taking part in a clinical trial was described as a way someone with cancer could be more in control of their health, indicating higher self‐efficacy, evident from engagement with and interest in their health.

I take an interest in my health, so I see being on a trial as a part of that.

(PID9)

#### 3.1.2 Theme 1.2: ‘Trusting This Is the Right Decision’

In deciding to participate, individuals considered many factors with the final decision perceived as stepping towards hope. For some, it was their *‘last hope’* (PID8) to access treatment. Where no other treatment options were available, clinical trial participation was an *‘easier decision’* (PID1), as *‘there was nothing to lose’* (PID17).

Whilst all participants said they understood the risks outlined in the participant information and consent form (PICF), these risks were outweighed by the possibility of a tumour response to treatment. Many reported feelings of trust in the doctor who referred them to the clinical trial, a major influence in their decision to participate. However, the consent process was reported as anxiety provoking, due to its formality and the amount of reading PICFs required. During the consent process there were many risks presented, and the prospect of living with multiple side effects acknowledged. *‘….well, I didn't really care what the side‐effects are, I will give anything a go basically’* (PID17). Many participants spoke of the information they received, saying they were clear on the potential risks from a clinical trial, even if they did not understand what those risks would look like. *‘How do you describe a sunset to a blind man?’* (PID 8). They did not consider uncertainty as a deterrent. Some described knowledge of side effects as a source of ongoing concern, which persisted for some time.

…there was a big wad of paperwork. He said you will either get the study drug or the placebo. He said there’s 36 side effects. I can remember those side effects. Every one. I never had any of those. So, it was a pleasant trial for me.

(PID15)

Many participants reflected on this period as a time when they came to understand the importance of trusting relationships with clinical trials staff.

[the] trial coordinator helps you stand up a bit straighter… I still didn’t know exactly what it was I going to feel or that would happen…she helped me through it.

(PID8)

### 3.2 Stage 2: Active Treatment

#### 3.2.1 Theme 2.1: ‘The Comfort of Structure’

Being on active treatment was described as predictable, bringing comfort and certainty.

…[it is] very organised which is comforting.

(PID14)

Protocolised assessments ensured care was provided according to plan and in a timely fashion. Knowing the schedule meant participants felt they knew what was happening, giving them a sense of control. Many spoke of falling into a routine, accepting a clinical trial took over many aspects of their lives. Some spoke of giving up hobbies, such as attending a local craft group, as their clinical trial appointments clashed with the weekly sessions. Others spoke of the travel required, up to 150 km round trip each time for some. Despite the negatives, knowing what to expect meant participants could draw on others outside the hospital to help, for example, neighbours to watch the farm or members of their church for transport. Many reflected on times they were not on a clinical trial and despite feeling unwell, having to quickly respond and organise treatment or navigate systems with little support. Many challenges diminished with a study protocol and additional resources with the clinical trial, resulting in improved experience of care overall.

…scans are always timely because of the protocol. Sometimes it is hit and miss when you are not on a clinical trial.

(PID9)

#### 3.2.2 Theme 2.2: Relationships With the Multi‐Disciplinary Team

While importance of health care team members involved in providing care was reflected throughout all stages, in the active treatment stage, it dominated many aspects of discussion. Across all interviews, participants indicated relationships with clinical trial coordinators, were very important, sometimes sustained over years. These relationships were often respected and terms such as *‘credible’, ‘professional’, ‘reliable’* used to describe clinical trials staff. Trust in the clinical trials team offset negative ideas about *‘big pharma’*. Other participants felt part of the clinical trials team. These relationships gave the clinical trial more value.

The blood tests weren’t coming back as they should. He rang someone, I got straight in for an appointment, we got things sorted out.

(PID2)

Relationships were also formed with others taking part in the same clinical trial.

…you just meet other people that have got the same sort of problem. We check on each other.

(PID14)

Some participants felt unsettled when unfamiliar clinical trials staff looked after them, feeling unseen, *‘just a list of boxes to tick’* (PID12).

#### 3.2.3 Theme 2.3: Developing Measures of Success

Participants described watching the clinical trials team responses as they performed assessments to gauge whether things were progressing in the *‘right direction’*, and for any indication of a treatment response. Many participants spoke of monitoring every clinical trial assessment and staff responses so they could measure their *‘fight’* against cancer. Each spoke of their own individual strategies to measure how treatment was going.

I was in pretty bad shape when I started. I’m pretty good now. Now eating and drinking, so it must be working.

(PID12)

The uncertainty of whether the trial treatment would be successful was prominent.

I had no idea how many people were getting the same sort of results as I did. And it would have been nice…someone must have a feeling or seeing all these different people as to how they’re reacting and be able to give some feedback somewhere.

(PID17)

If there was a reason participants could not remain on trial treatment, it was perceived a setback. Consequently, side effects were concerning, and how well individuals coped with symptoms had an impact on their management of treatment and uncertainty. Some spoke of doing all they could to stay on treatment, considering remaining on treatment a marker of success.

The thought of treatment breaks can be scary.

(PID4)

### 3.3 Stage 3: Life After Treatment

#### 3.3.1 Theme 3.1: Finding ‘Meaning’ From Taking Part in a Clinical Trial

Finishing treatment was cause for celebration. Ongoing monitoring after the clinical trial treatment ended brought ongoing value and reassurance.

I mean, all they did is give you a pill and look after you every so often. But probably that’s the difference. Not the pill giving, but the monitoring. And that’s been a comfort.

(PID15)

All participants who had finished active treatment reflected on how their illness and clinical trial participation may benefit others.

…so the drug did not help me like I thought, I’ve got spots on my liver now. I’ve been inundated with abscesses every three or four months…but I cannot think of one bad thing that came from the clinical trial.

(PID3)

Some proposed taking part in a clinical trial was a responsibility that *‘…one must take’* (PID12), *‘…a way to do something to help’* (PID 15). Additionally, participants felt they had become experts in their own right. Many valued their experience and having knowledge to share with others about clinical trial participation.

…if someone’s asking about clinical trials, I can tell them about Phase 1, Phase 2, Phase 3, and about whether it’s blind study or whether it’s this or that.

(PID6)

As participants made sense of their clinical trial journey, it was the relationships that anchored them through each step.

#### 3.3.2 Theme 3.2 ‘Staying Close to the Mother Ship’

Participants wanted to remain connected to their treatment centre, the clinical trials health care team and the clinical trial itself, even after completing treatment. There were common frustrations that came with taking part. These included frustration with lack of car parking and transport, and the high financial costs associated with these were common negative experiences. Some participants expressed frustration about wait times for appointments. This was largely due to the additional assessments required for a clinical trial, if each appointment was running one or two hours behind, it made for a very long day. These concerns were felt more acutely when participants had completed treatment. Some spoke of leaving home at 5a.m. to be sure they could get a carpark but happy ‘*put up with it*’ (PID 15). Tolerating these frustrations was presented as a personal contribution to the study and something they accepted to stay connected.

Participants reported a sense of investment which came from taking part in the clinical trial. They wanted to know how the clinical trial was progressing, desiring regular updates. Questions participants had, included: how many people were participating at their site, and across Australia; was the study happening in other countries; how long it would run; and, if there were people just like them. Participants also spoke of wanting to know the trial outcomes to see how they may have helped others. Participants used a variety of information sources to learn about trial outcomes, including reports from their original referring team (who also wanted to know the results but weren't sure). Some participants spoke of searching the internet to find out the results, hearing news from support groups, or other news sources.

I saw in the paper three weeks ago, and they said the study has been a hit and it has extended people’s longevity.

(PID 16)

### 3.4 What This Study Tells Clinical Teams and Health Services

**FIGURE 1:**
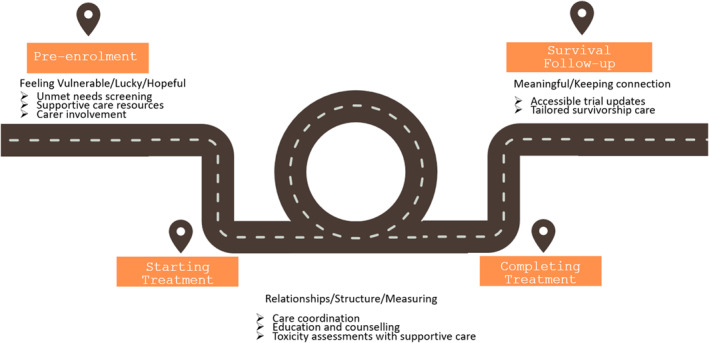
An Infographic of the clinical trial pathway developed using an interpretive analysis. The pathway presents concepts relating to pre‐enrolment, starting treatment, completing treatment and during survival follow‐up. Potential supportive care strategies are proposed at each point in the journey.

The key learnings for clinical teams and health services are presented in Figure 1, drawing on the interpretative analysis findings. In essence, the needs of patients were dependent on the stage of the clinical trial.

## 4 Discussion

We aimed to explore the experience of participating in an ANZUP urogenital or prostate clinical trial. Variable experiences of participants according to the stage of the clinical trial showed that participating in a clinical trial is a journey. In the early stages (prior to enrolment), this was a time of considerable uncertainty. Despite this experience, participants believed choosing to participate was a decision to take positive action. A time of stability and feeling in control came when treatment started. Treatment toxicities were expected but avoided, or managed, and the trial provided clear expectations of appointments and commitments. Life after treatment was seen as a time to reflect and take stock, whilst remaining close to those who had cared for them during a time of need.

Our study was novel in that it asked questions specific to the stage of a clinical trial and identified that experiences can be determined by these. Studies that discuss experiences at a specific stage of a trial support our study findings, but research outside of pharmaceutical industry sponsored trials is limited. In an ANZUP study recruiting a different cohort of participants for the TheraP study, authors recommended ongoing supportive and psycho‐oncology care during any clinical trial [23]. In our study, the screening process was a time when participants felt most unsettled, and the literature reports a need for patients to feel safe as they weigh up their participation in a clinical trial [23]. It is recommended information resources are available during this time to ameliorate the sense of sitting in a holding pattern [13]. It is possible these experiences reflect the deviation from standard of care pathways in health systems, including the supportive care infrastructure, highlighting a need to better integrate any clinical trial pathway with available supportive care resources as much as possible, such as distress screening, psycho‐oncology, specialist nursing and palliative care. The interpretative analysis findings provided guidance on what could be planned prior to site initiation. One commonly used strategy is to map a clinical trial protocol against local procedures and processes. Such an approach may optimise patient experience, support patient safety and further formalise integration and implementation of clinical trial procedures within existing institutional practices.

The period during active treatment was described as a time of normalcy and structure for clinical trial participants, also consistent with the broader literature. We were not able to find any research, however, that spoke specifically of participant experience after treatment had ended, with those in our study speaking of their personal investment in the clinical trial. With some clinical trials running for many years, waiting until the end of the trial to learn the outcomes can be challenging. There is strong evidence that identifies the importance of providing updated information resources during clinical trials [9] and opportunities to have input into trial conduct [7, 24], ensuring acceptability to both patients and their clinical teams. It is challenging to balance this with the regulatory limitations of what information can be made available, but study sponsors can provide support through regular trial updates through social media and a web presence made known through the Participant Information Sheet.

Another novel finding, albeit present in non‐clinical trial research, we found the relationships with health teams featured strongly, described as reliable, available, transparent, and accepting of patient choices [2, 19, 24]. Participants spoke of trusting the person who referred them to the trial as having their best interests at heart. It has been demonstrated that a lack of trust is a common reason for participants to withdraw from a cancer clinical trial [25]. During treatment, clinical trials staff helped participants navigate appointments and understand side effects. When treatment ended, staying connected for individualised systematic monitoring was a key role of clinical trials staff. These findings also align with the wider literature, where studies report trust is built by rapport, compassionate care, and honesty [26], engendered by meeting a need and showing respect for a patient's wishes [27]. Research on using ethical frameworks report that trust is relational and mutual [28, 29, 30, 31].

Led by the pharmaceutical industry, structures are emerging to incorporate measurement of patient experience into the operationalisation of clinical trials day to day [32, 33, 34]. Whilst these are being tailored to different health contexts [34], there is a lack of published information sharing the outcomes of using these. Future work is needed to understand if there is a benefit, not only the clinical trials community, but for patients and supports, and health services more generally.

### 4.1 Clinical Implications

Interestingly, despite wanting to understand the experiences of those who have participated in an ANZUP investigator led urogenital or clinical trial, our findings could be applied to any cancer type, as a part of standard care or a clinical trial intervention. This is despite the fact that those choosing to participate in a clinical trial may differ from the wider population diagnosed with cancer.

The results of this study highlighted that those participating in a clinical trial have unique needs prior to treatment, including access to supportive care for both patient and carers, including distress screening, and tailored information resources. During treatment, unique needs were identified relating to supportive care, care coordination, and education and counselling. After treatment completion, access to trial updates and survivorship care were identified as unique needs. Future clinical trials may improve patient experience if they incorporate these needs into protocol design, and if assessments also align with standard of care supportive care service provision.

### 4.2 Study Limitations

The participants were recruited by clinical trials staff who supported ANZUP studies, and it is possible that this may have influenced the findings reported. However, our study sequentially approached patients to participate in order to reduce the possibility only those with a positive clinical trial experiences were interviewed. Participants were interviewed over 6 months in June 2019 to December 2019. One participant reported withdrawing from a clinical trial when not randomised to their preferred treatment but still participated in this qualitative study.

## 5 Conclusions

Participants on a clinical trial journey can have a range of experiences according to the stage of the clinical trial. Integrating clinical trials with routine care in health services may ensure patient wellbeing and safety.

### 5.1 Positionality Statement

When the research manuscript was drafted, two authors identified as women and five as men. One was a nurse who had previously worked as a clinical trial coordinator and the other female was psycho‐oncologist and clinical trials investigator. Three men were clinical trials investigators, and two men were health students.

## Consent

All participants provided written informed consent prior to participating in interviews.

## Supporting information

Supporting Information S1

Supporting Information S2

## Data Availability

Data will be made available upon request due to ethical/privacy reasons.
