# Supplementary material for: How to Support Participants in Urogenital and Prostate Clinical Trials: A Qualitative Perspective
Source: Psychooncology. 2025 Jul 14;34(7):e70214. doi: 10.1002/pon.70214 (PMC12260335; doi:10.1002/pon.70214)
Supplement: Supplementary file 2 — Supporting Information S2 [file PON-34-e70214-s001.docx]

Supplementary File 1: Qualitative Interview Guide

1. Clinical trials awareness

Before your cancer diagnosis had you heard of clinical trials or clinical research? What did you know about research at that time?

How has your knowledge changed?

1. Deciding to take part in a clinical trial

How did you come to take part in a clinical trial?

Is there anything that comes to mind when think about participating in a clinical trial?

How did you decide whether you would take part?

Did anyone help you with that decision?

Do you remember when you first heard about the clinical trial?

What happened when you signed the consent form, what stands out in your memory?

1. Clinical trial procedures

In the clinical trial you participated in, did you go through a process where you were allocated to a particular treatment schedule?

Do you remember starting on the trial?

What did you remember about the first day?

How did/is your treatment go/going?

Have you had any problems or side effects? (If yes) How did you manage those? What about the health team?

How did you go with the study assessments? Did you have much to do? How did you organise your time and appointments? Is there anything you would do differently to organise the scans, blood tests and paperwork?

Did you have to learn any new skills? How did that go?

1. Finishing treatment

(if applicable) Do you remember finishing treatment?

(if applicable) How are you going now that you have finished treatment?

1. Results of trials

Have you heard about any results from the study? Have you got any thoughts about this?

1. Thoughts about clinical trials

How do you think clinical trial participation is different to other treatments?

Would you recommend taking part in a clinical trial to other people? Why?

What is the worst part about taking part in a clinical trial? Why?

What do you think is the best thing about taking part in a clinical trial? Why?

What has really helped with taking part in a clinical trial?

Is there anything you would change?
